# TIME Is a Great Healer—Targeting Myeloid Cells in the Tumor Immune Microenvironment to Improve Triple-Negative Breast Cancer Outcomes

**DOI:** 10.3390/cells10010011

**Published:** 2020-12-23

**Authors:** Swarnima Singh, Xiang H. F. Zhang, Jeffrey M. Rosen

**Affiliations:** 1 Department of Molecular and Cellular Biology and Dan L Duncan Comprehensive Cancer Center, Baylor College of Medicine, Houston, TX 77030, USA; swarnims@bcm.edu; 2 Translational Biology and Molecular Medicine, Baylor College of Medicine, Houston, TX 77030, USA

**Keywords:** breast cancer, macrophage, triple-negative breast cancer, myeloid-derived suppressor cells, immune

## Abstract

The word myeloid is derived from the Greek word *muelós* which means “marrow”. Therefore, myeloid cells are described as cells that arise in the bone marrow. They can be distinguished from lymphoid cells based on their different differentiation trajectories—Lymphoid cells (B and T cells) are usually born in the bone marrow, but they need to migrate to lymphoid organs to mature and differentiate usually in response to antigens produced due to infections and diseases like cancer. On the other hand, myeloid cells do not follow this differentiation trajectory. They arise from the bone marrow, and do not need an encounter with antigens to gain their functionality. Thus, while lymphoid cells are a part of the adaptive immune system, myeloid cells are a part of the innate immune system. Hematopoiesis gives rise to two progenitor cells—the common myeloid progenitor (CMP) and the common lymphoid progenitor (CLP). The CMP can give rise to megakaryocytes, erythrocytes, mast cells and myeloblasts. Myeloblasts in turn lead to the formation of basophils, neutrophils, eosinophils and monocytes that can further differentiate into macrophages. This review will focus on macrophages as well as the phenotypes they acquire with the tumor immune microenvironment (TIME) in triple-negative breast cancer (TNBC). It will address how cancer cells in the tumor microenvironment (TME) recruit macrophages and may switch to recruiting neutrophils upon depletion of these tumor-associated macrophages (TAMs). Finally, it will also shed light on past and current treatment options that specifically target these cells and how those affect patient outcomes in TNBC.

## 1. Introduction

Phagocytes (Macrophages) were first described by Russian zoologist Élie Metchnikoff with his experiments on starfish larvae. Metchnikoff showed that introducing citrus thorns into the larvae of starfish resulted in an unusual accumulation of white blood cells. He hypothesized that these white blood cells were attracted to sites of inflammation and could phagocytose bacteria, which led him and other scientists to name them phagocytes [1].

The prevailing dogma since the 1960s, as put forth by Van Furth and colleagues, was that under normal conditions, tissue-resident macrophages arose from circulating blood monocytes that formed in the bone marrow [2]. However, subsequent studies have shown that tissue-resident macrophages usually originate from the embryo sac or fetal liver during embryonic development instead of the bone marrow [3,4]. These cells can self-renew, however certain tissue-resident macrophages such as the peritoneal macrophages are constantly replaced by circulating blood monocytes over time [5]. Furthermore, both bone marrow-derived, and tissue-resident macrophages can be reprogramed depending on the microenvironment in various normal tissues.

Tissue-resident macrophages are specialized phagocytes that migrate to sites in response to inflammation or injury to phagocytose debris and facilitate wound healing. Interestingly, normal macrophages can even present antigens. However, they lack the ability to migrate to lymphoid tissues in large numbers to activate T cells as compared to professional antigen-presenting cells such as dendritic cells (DCs) [6].

## 2. Macrophages in Normal Mammary Glands

The mammary gland (MG) is an organ whose differentiation occurs primarily postnatally. It is derived from the epidermis around E10.5 and after the formation of buds, rudimentary ducts are present by E18 [7]. Recent studies have shown that fetal liver and yolk sac derived macrophages are present in the MG around E16.5 challenging the widespread notion that MG macrophages are derived from bone marrow and recruited to the terminal end buds (TEBs) postnatally [8]. During mammary gland remodeling from birth through puberty, macrophages assist in phagocytosing dying epithelial cells and in maintaining the underlying structure of the MG. Recent studies have shown that ductal macrophages in the normal MG are a unique population that differ from stromal macrophages. They are more similar to tumor-associated macrophages (TAMs), showing that different microenvironmental conditions can indeed reprogram macrophages to perform specialized functions within the tissue [9].

## 3. Triple-Negative Breast Cancer

Triple-negative breast cancer (TNBC) is a subtype of breast cancer that is defined by the absence of the estrogen and progesterone receptors and HER2, which provide specific drug targets for those subtypes [10]. If it were considered a distinct disease, TNBC would rank as the fifth leading cause of cancer deaths in women. It is an aggressive and hard to treat form of breast cancer with higher rates in younger women and women of African-American ancestry and cytotoxic chemotherapies are often the mainstays of treatment [11].

Most research has focused on TNBC tumor cells and the tumor microenvironment (TME) and how the interactions between tumor and stromal cells lead to chemo-resistance and increased metastatic potential. However, with the recent success of immunotherapy, there has been increased interest in the tumor immune microenvironment (TIME) and how to reprogram it to facilitate tumor regression in both the primary and metastatic sites. A form of immunotherapy that has been a success in many different cancer types such as melanoma and non-small cell lung cancer is checkpoint blockade. These therapies work by targeting inhibitory signals on T cells such as PD1 and CTLA4. The inhibition of these signaling molecules on T cells can lead to the reinvigoration of these T cells allowing them to mount effective anti-tumor responses [12,13].

The combination of anti-CTLA4 and PD1/PDL1 therapies have led to remarkable responses in certain tumors. When compared to monotherapy, combination therapy can induce around 22% complete response (CR) in patients with metastatic melanoma [14]. Combination therapy can also lead to an improvement of overall survival in patients with renal cell carcinoma as compared to the standard-of-care monotherapy sunitinib [15]. The responses to combination immunotherapy have varied in TNBC or metastatic TNBC and have not shown the remarkable efficacy that is seen in metastatic melanoma. The clinical trial IMpassion131 combined an anti-PD-L1 antibody atezolizumab in combination with paclitaxel, which is a cell cycle inhibitor in patients with metastatic or non-resectable TNBC. A cohort of 651 patients were enrolled in the phase III trial, however, no improvement in progression-free survival (PFS) or overall survival (OS) was seen in the combination group vs. paclitaxel alone group [16]. IMpassion130 however, showed an improvement in OS when a nanoparticle albumin-bound form of paclitaxel (nab-paclitaxel) was used in combination with atezolizumab in an interim analysis [17], however while was no clear benefit in OS in the final analysis of the trial, clinicians saw a meaningful survival benefit in PDL1 positive patients [18]. The results of these trials highlight the importance of using drugs that have improved pharmacokinetics and pharmacodynamics to treat solid tumors as well as biomarkers to pick cohorts of patients that would benefit the most. The I-SPY2 neoadjuvant trial was conducted in patients with grade II/III TNBC. In this trial, the response to a standard-of-care neoadjuvant chemotherapy (NACT) was compared to a combination of PD1 inhibitor pembrolizumab and NACT. The addition of pembrolizumab to chemotherapy had remarkable effects leading to a dramatic improvement in the rates of patients that had a pathologic complete response (pCR). pCR rates were 44% vs. 17%, 30% vs. 13% and 60 % vs. 22% in the three cohorts of combination therapy vs. NACT alone in treated patients that have been evaluated so far [19]. While these response rates are encouraging so far, a key question still remains—“Why are the remaining~50% of patients not responding to an immunostimulatory drug combined with a highly cytotoxic chemotherapy regime?”. One aspect of TNBC biology that is apparent here is that there are distinct subtypes within TNBC that need treatment options beyond chemotherapy and current immunotherapies. Additionally, subsets of TNBC with differing immune infiltrates might also exhibit differential responses to therapies.

## 4. The TIME and T Cells

T cells are major players in the anti-tumor immune response. Studies have shown that the presence of tumor-infiltrating lymphocytes (TILS) especially CD8+ and CD4+ T cells within the TME lead to better prognosis and response to chemotherapy in TNBC patients [20]. The localization of TILs can affect prognosis in TNBC as well. Tumors can be divided into two main groups based on CD8+ T cell infiltration. Group 1 has very low numbers of T cells in the core of the tumor and if they do have T cells these are restricted to the margins of the tumor [21]. These are known as “immune desert” tumors and they also have an enrichment for signatures associated with fibrosis. The tumors that do have a high CD8+ T cell infiltration to the core can further be subdivided into two categories—tumors where the T cells are restricted to the tumor stroma and tumors where there is an enrichment of CD8+ T cells in the tumor cell compartment. They have also derived signatures from these tumors that show that tumors with a low immune infiltrate and a high fibrotic signature have a significantly worse prognosis as compared to tumors with a high immune and low fibrosis signature [21].

The TIME is a dynamic environment that interacts with tumor cells as well as stromal cells present within the tumor. These interactions can affect responses to treatment by helping tumor cells evade cytotoxic immune cells by downregulating the expression of tumor-specific antigens thereby facilitating tumor cell escape to form distant metastases. An important non-cellular aspect with the TIME is the extracellular matrix (ECM). Cells within the ECM such as cancer-associated fibroblasts can release chemokines and cytokines such as Cxcl12 that can recruit CD25+ Foxp3+ T regulatory cells to inhibit effector T cell functions and lead to T cell exhaustion [22]. This reduced T cell infiltration is a major mechanism of resistance to chemotherapy. Recent studies have shown that the suppression of transforming growth factor-β receptor 2 (TGFBR2) in CD4+ T cells has potent anti-tumor effects. These include an increase in hypoxia-associated tumor cell death by remodeling the leaky tumor vasculature into a more organized structure. This study challenges existing notions of the pro-tumor roles of type 2 IL4-mediated CD4+ T cell responses and underscores the importance of further detailed mechanistic studies in the TNBC TIME [23].

TAMs are highly immunosuppressive and one of the ways they accomplish this is by inhibiting anti-tumor T cells. Cutting-edge imaging studies done in slices of fresh human lung squamous cell carcinomas have shown that stromal TAMs can trap CD8+ T cells via physical contact that prevents them from reaching tumor islets. Tumors with a high infiltration of TAMs have T cells with impaired motility [24]. CSF1R is a TAM recruitment and differentiation factor acting through the CSF1/CSF1R axis [25]. In vivo experiments done in the mouse mammary tumor virus (MMTV)-PyMT model of breast cancer have shown that the depletion of stromal TAMs using a CSF1R inhibitor (PLX3397) can increase T cell motility and the average lengths travelled by these cells as well as increase the number of T cells in the tumor islets. This increased motility was due to the inhibition of physical interactions between T cells and stromal TAMs rather than a remodeling of the ECM after treatment. The investigators also showed that the combination of PLX3397 and an anti-PD1 antibody had a strong inhibitory effect on tumor growth in multiple models [24]. Therefore, there is an urgent need to develop and test therapeutics that go beyond the current immunotherapies that can target key immunosuppressive players such as TAMs within the TIME.

## 5. Tumor-Associated Macrophages in Breast Cancer

Tumor-associated macrophages (TAMs) are macrophages that infiltrate the tumor and its microenvironment. There they enable tumor growth and metastasis by a variety of mechanisms such as promoting blood vessel formation (angiogenesis), suppressing cytolytic T cell responses and mediating chemoresistance [26]. There are two types of macrophages that promote tumor cell intravasation. Migratory macrophages can facilitate tumor cell movement towards blood vessels in the MMTV-PyMT model of breast cancer by forming physical contacts through an EGF/CSF1 paracrine loop. A subset of these cells can also differentiate into perivascular TAMs that promote vascular leakiness and intravasation of cancer cells [27]. Indeed, tumors cells that co-migrate with TAMs have been shown to be more successful in reaching distant sites and creating metastases [28].

TAMs are believed to originate in the bone marrow as monocytes which are then recruited to the primary tumor via the CCL2/CCR2 signaling axis [29]. However, studies have shown that even tissue-resident macrophages derived from the YS or FL can be reprogrammed in-situ by signals derived from tumors cells. For example, primary pancreatic ductal adenocarcinoma cells (PDAC) can release small extracellular vesicles known as exosomes that can home to the liver, recruit Kupffer Cells and create an environment favorable for liver metastasis [30].

Monocytes within the tumor can differentiate into different subtypes of macrophages that have been classified into three distinct states that have been well characterized in vitro, namely, M0, M1 and M2. M0 macrophages are induced by tumor cell-secreted colony stimulating factor 1(CSF1). Due to microenvironmental cues these resting macrophages can skew into two extreme phenotypes designated M1 and M2 macrophages. These represent extreme ends of a functional spectrum with a number of intermediate phenotypes [31]. These phenotypes are harder to induce in vitro and have mostly been characterized using in vivo studies. Studies have also shown that the breast tumor microenvironment can educate macrophages and skew them to an “M2”-like phenotype [32].

M1 macrophages are known as classically activated macrophages. They are induced by factors such as IFNγ or TNFα that are secreted by a variety of cells including cytotoxic CD8+ T cells, and they can elicit antitumor effects in a variety of ways including direct tumor cell killing. They are proinflammatory, can secrete factors such as IL12, CXCL9 and CXCL10 that can recruit T cells, and are also efficient antigen-presenting cells (APCs).

M2 macrophages are at the opposite end of the spectrum. Induced by the expression of cytokines such as IL4, these cells are anti-inflammatory and immunosuppressive. These cells are highly phagocytic, can modulate the ECM by depositing substrates such as collagen that hinder T cell motility and also can induce a strong protumor response. They accomplish this by secreting chemokines such as CCL17, CCL22 and CCL7 which can interact with receptors such as CCR3 and CCR4 that are preferentially expressed on immunosuppressive T regulatory cells [33]. They can also promote tumor angiogenesis via the production of growth factors such as Il8, EGF and VEGF. Studies done on invasive breast carcinoma patient samples have shown that breast TAMs produce CCL18 which can increase the adherence of cancer cells to the ECM and promote migration [34]. Additionally, they can promote resistance to chemotherapies such as Paclitaxel by disrupting mitotic arrest in breast cancer cells and leading to increased cell proliferation and viability [35].

M1 and M2 TAMs are metabolically distinct. The changes in the metabolic profiles are mainly induced by cues within the TME which enable TAMs to switch from a dependence on glycolysis to oxidative phosphorylation. M1 macrophages are mainly dependent on glycolysis for their ATP needs and studies have shown that inhibition of ATP can dampen antitumor responses in these cells, reduce reactive oxygen species (ROS) as well as decrease the release of several pro-inflammatory cytokines. In contrast, M2 macrophages have an intact Krebs cycle and are dependent on fatty acid oxidation (FAO) and oxidative phosphorylation (OXPHOS) to fulfill their ATP requirements. With intact OXPHOS signaling, M2 macrophages produce lower amounts of reactive oxygen (ROS), signaling their departure from a pro-inflammatory M1 macrophage phenotype [36,37].

The advent of high dimensional sequencing technologies such as single-cell RNA sequencing has further advanced our understanding about TAM heterogeneity in certain cancers. Studies done on TAMs in gliomas have shown transcriptomic differences in tissue-resident and circulating TAMs as well as an upregulation of the TCA cycle in blood-derived TAMs that also exhibit enhanced phagocytic potential [38]. Interestingly, the blood-derived TAMs also showed an increased propensity for oxidative phosphorylation, which is commonly known to be associated with “M2” TAMs; however, they co-expressed both M1 and M2 TAM markers. There has similarly been a lot of interest in TAM heterogeneity in TNBC and preclinical murine models are proving to be important tools in further understanding the different functions of TAM subsets in breast cancer.

## 6. Preclinical Models of TNBC

Mouse models play an integral role in understanding the TIME in TNBC. Cell-derived xenografts (CDX) and patient-derived xenografts (PDX) are essential in understanding the biology of tumor/stromal cell interactions to elucidate the cell intrinsic mechanisms that govern treatment resistance and promote metastasis. Additional advantages of PDX are that they may recapitulate the genomics and phenotypes of the original patient tumors, therefore making these very attractive for testing drug responses and predicting sites of future metastases. However, there are some drawbacks to using these models. CDX and PDX are implanted in severely immunocompromised genetically engineered mice, such as the NOD/SCID mouse [39], that either lack functional T/B cells or lack a thymus which in turn leads to a depletion of T cells. So, while they are appropriate models to study tumor and stromal cells, they lack most of the players in the adaptive immune system that are essential in promoting long-term tumor responses.

Syngeneic mouse models for TNBC include cell-based models such as 4T1 that can be orthotopically implanted into immunocompetent Balb/c mice [40]. These are useful to study the dynamics of the immune system in response to drug treatment and their roles in facilitating metastases.

Genetically engineered mouse models (GEMMs) are another useful tool to model TNBC in immunocompetent mice. One of the most commonly known models to study breast cancer is the MMTV-PyMT model in which the long terminal repeat (LTR) sequence of the mouse mammary tumor virus (MMTV) drives the expression of the polyomavirus middle T antigen specifically in the mouse mammary gland. Studies showed that the expression of the middle T antigen could transform the mammary epithelium and result in primary mammary adenocarcinomas as well as lung metastases [41]. Additionally, the MMTV-PyMT mouse model could recapitulate the various stages of cancer progression in human patients starting from normal breast-like to ductal hyperplasia and ductal carcinoma in situ (DCIS) and finally multifocal malignancies similar to human invasive ductal carcinoma [42]. Researchers crossed the MMTV-PyMT mouse to a Csf1op mouse that had CSF1-null recessive mutations. This resulted in a mouse that lacked CSF1R which maintains the proliferation and differentiation of monocytes and TAMs. Interestingly, the absence of CSF1 did not have a direct effect on tumor growth in the primary setting, but it did show a delay in the formation of lung metastases. Transgenic expression of CSF1 resulted in a huge influx of TAMs into the primary tumor and led to accelerated growth and significantly increased the lung metastatic burden [43]. This was one of the first studies to show the involvement of TAMs in promoting metastasis. Further studies done by these investigators and their collaborators defined the role TAMs played in consort with tumor cells to promote angiogenesis and accelerate tumor progression [44].

P53 is a known tumor suppressor and mouse models with mutations in the p53 gene are prone to developing spontaneous lymphomas and leukemias, but rarely develop breast cancer, unlike patients with the Li–Fraumeni Syndrome. Studies have shown that around 80% of triple-negative basal human breast tumors have missense or nonsense mutations in the p53 gene [45]. Additionally, loss of heterozygosity or mutations in p53 have shown clear associations with reduced cytotoxic CD8+ T cell infiltration in patients with triple-negative or basal-like breast cancers [46]. Studies done in multiple GEMM models of breast cancer including TNBC have shown that the loss of p53 has resulted in an increased secretion of IL-1β by TAMs through the Wnt signaling pathway that in turn results in the systemic recruitment of neutrophils and increased metastatic capability [47].

To generate p53 null murine models of breast cancer, researchers transplanted the mammary epithelium of germline p53 null mice into the cleared fat pads of wild-type Balb/c females. The Balb/c mice were much more susceptible to radiation-induced breast cancer than the more commonly studied C57BL/6 strain. The outgrowths were retransplanted for 10 generations and resulted in multiple tumors that were histologically distinct [48]. Subsequent gene expression profiling of these tumors showed that they clustered with the known human subtypes of breast cancer—luminal, basal-like and claudin-low [49].

Claudin-low tumors are characterized by the low expression of claudins 3 and 7 and a high expression of mesenchymal markers such as Slug, Snai1, Zeb1 and Vimentin. Additionally, these cells are highly infiltrated by TAMs and have low numbers of tumor-infiltrating neutrophils (TINs). These highly aggressive tumors are representative of patient TNBC [50]. Results from the ARTEMIS trial at M.D Anderson Cancer Center also have shown that patients with TNBC that have a high TAM infiltrate and EMT signature have low rates of response to neoadjuvant chemotherapy [51].

## 7. MES to NES in TNBC

**Figure 1:**
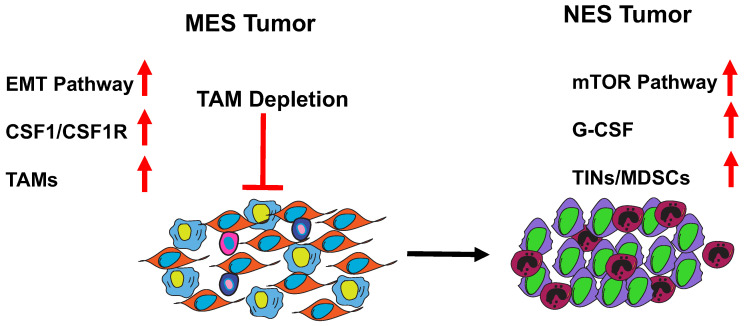
Macrophage (MES) tumors can switch to neutrophil (NES) tumors upon depletion of tumor-associated macrophages (TAMs). The depletion of TAMs in macrophage-enriched tumors by using CCR2-/- mice and a CSF1 neutralizing antibody alongside liposomal clodranate results in an interesting phenotypic switch in these tumors leading instead to the recruitment immunosuppressive neutrophils (Figure 1). Resistance to checkpoint blockade in these tumors is also mediated by the accumulation of neutrophils. Depleting these neutrophils by using a Ly6G neutralizing antibody in combination with checkpoint blockade led to a significant reduction in tumor recurrence [52].

The TIME is a highly dynamic environment. It can remodel itself in response to treatments as well as during progression and metastasis. Researchers have studied multiple models of murine TNBC as well as clinical data sets and identified tumors that are either macrophage (MES)- or neutrophil (NES)-enriched and have a number of strategies to evade cytolytic T cells and promote tumor progression (Figure 1, [52]).

Neutrophils are a part of the innate immune system as well. These originate in the bone marrow from the CMP in response to infection or to cues from tumors. They are short-lived cells that are phagocytic in nature and often are the body’s first defense against infections where they are followed by macrophages that can persist for longer periods of time. Within the tumor microenvironment, tumor-infiltrating neutrophils (TINs) can suppress T cell functionality and promote the formation of pro-tumor CD4+ T regulatory cells [53]. TINs that have the ability to suppress T cell proliferation and responses in vitro and in vivo and are also called myeloid-derived suppressor cells (MDSCs). These cells are heterogenous in nature consisting of immature progenitor cells as well as more terminally differentiated cells. MDSCs in mice that express markers such as Ly6G are known as granulocytic MDSCs (gMDSCs) and they are enriched in certain murine models of breast cancer and can drive the survival and proliferation of tumor-initiating cells (TICs) [54]. Another phenotype of MDSCs is monocytic MDSCs (mMDSC). These are marked by high expression of Ly6C and a low expression of Ly6G. Again, these might represent the opposite ends a continuum of intermediate states, but more studies are needed to better understand MDSC heterogeneity with the TIME.

## 8. Role of MDSCs in Breast Cancer and Metastasis

MDSCs are known to cause the inhibition of multiple anti-tumor immune cell-types such as T cells, NK cells and even dendritic cells. They are recruited to primary breast tumors by a number of factors such as G-CSF, IL6, IL7, IL12, IL10 and IL1b. They can also be recruited to the TME via the CCL2 signaling axis [55]. A study in prostate cancer showed MDSCs were recruited by a YAP1-mediated CXCL5–CXCR2 signaling axis and that the inhibition of YAP led to a decrease in the accumulation of MDSCs. Blocking CXCR2 on MDSCs has shown to increase the efficacy of tumor regression in CCR2 KO mice [56].

**Figure 2:**
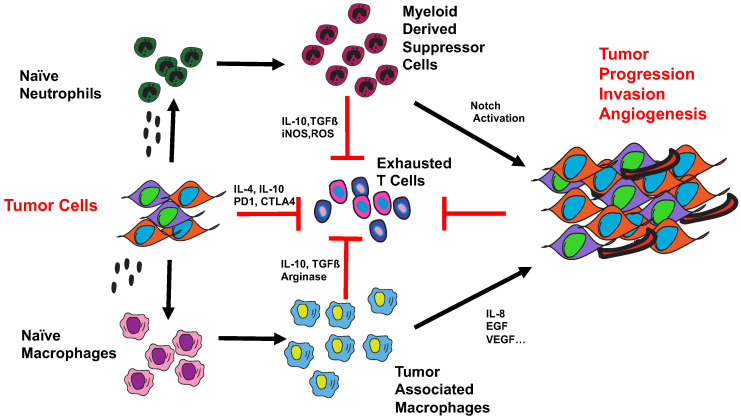
TAMs and tumor-infiltrating neutrophils (TINs) can promote tumor progression and metastasis by inhibiting anti-tumor T cells and promoting the survival of tumor-initiating cells (TICs).

MDSCs can secrete a number of factors that directly affect tumor cell survival and metastasis by secreting immunosuppressive factors such as TGFß and IL10. These factors can skew TAMs into an “M2”-like phenotype which has been shown to have potent pro-tumor effects and induce the expansion of Th2 and T regulatory (Treg) cells [57]. They can create immunosuppressive conditions for T cells in a number of ways. They produce iNOS that can directly affect the nitrosylation of the TCR complex and impair signaling and activation as well as affect the recruitment of T cells by nitrosylating CCL2 [58]. They can also secrete reactive oxygen species (ROS) and deplete the local TME of arginine and citruline which are key nutrients for T cells, therefore limiting T cell proliferation and differentiation as highlighted in Figure 2 [59]. Studies have shown that MDSCs can promote angiogenesis and promote metastasis of tumor cells by inducing an epithelial-to-mesenchymal transition (EMT) in tumor cells thereby facilitating their escape from the primary site to distant organs [60].

## 9. Clinical Relevance and Biomarkers for TAMs and MDSCs

Studies done on invasive breast carcinoma patient tissue samples have shown that high expression of CCL18 in TAMs correlates with metastasis. Additionally, patients with high levels of CCL18 have a significantly poor prognosis [34]. CD68 is often used as a TAM marker in patients. An increase in CD68+ TAM infiltration is associated with higher grade breast cancers and as well as shorter disease-free survival (DFS) and overall survival (OS) in TNBC [61].

Clinically, the presence of residual circulating gMDSCs (CD11b^+^CD33^+^HLA-DR^−^CD15^+^ cells) after NACT has been associated with poor survival and an increased chance of metastasis in patients with TNBC. mMDSCs (CD14^+^HLA-DR^low/−^CD86^low/−^CD80^low/−^CD163^low/−^) are significantly elevated in the peripheral blood of patients with breast cancer, especially those with locoregional or distant metastasis [62]. Additionally, high TIN scores in melanoma patients are also associated with progressive disease (PD) [63]. Preclinical studies have identified CCL9+ gMDSCs in the primary breast tumor-mediated lung premetastatic niche. Furthermore, the knockdown of CCL9 can reduce tumor volumes and reduce the metastatic load in murine models of metastatic breast cancer. The human orthologue of CCL9 is CCL23 and studies have shown the expression of CCL23 in the peripheral blood mononuclear cells (PBMCs) is associated with PD [64].

## 10. Therapeutic Interventions for TAMs and MDSCs

Due to extensive preclinical studies of TAMs in mouse models, there are several treatment options that are being tested in clinical trials (Table 1). These either work by inhibiting different signaling pathways in these cells or by skewing these cells into an anti-tumor phenotype. Some of the pathways being targeted are as follows:

1. CSF1-CSF1R Inhibition—Colony stimulating factor receptor 1 (CSF1R) is a receptor for colony stimulating factor 1 (CSF1). Multiple drugs are being studied in preclinical and clinical studies to determine the therapeutic benefits of inhibiting this axis. For example, PLX 3397 is a receptor tyrosine kinase inhibitor of CSF1R. Preclinical murine models have shown that the drug can inhibit TAMs and improve T cell infiltration into the tumor islets in a murine mammary cancer model [25].

2. MerTK—MerTK is a phagocytic receptor expressed by macrophages. It is involved in the clearance of tumor cell debris by macrophages. Targeting MerTK has resulted in the accumulation of apoptotic bodies within the TME and resulted in a type I interferon response. The inhibition of MerTK also synergized with anti-PD1 antibodies in a murine model of colorectal cancer and led to a significant decrease in tumor volume [65].

3. CCL2/CCR2—The CCR2/CCL2 signaling axis is key to the recruitment of bone marrow-derived monocytes and macrophages into the TIME. Depletion of CCR2 in murine models has shown a decrease in the number of TAMs as well as an increased responsiveness to checkpoint inhibitors and a decrease in metastasis [66]. Pharmacological inhibition of this pathway includes drugs such as PF-6309 that is a small molecule antagonist of CCR2 currently being studied in a Phase 1b/2 trial for pancreatic cancer.

4. CD47-Sirpα—CD47 is a transmembrane protein expressed on the surface of many cells including tumor cells and helps in the survival of these cells. It can bind to Sirpα that is expressed by phagocytic macrophages to prevent them from phagocytosing tumor cells [67].

5. CD11B—CD11B is an integrin expressed on the surface of myeloid cells including TAMs and TINs. It is involved in cell migration and recruitment and hence is an attractive therapeutic target to prevent tumor cells from recruiting immunosuppressive cells. Blocking CD11b in humans using antibodies or small molecules has yielded disappointing results as a very high dose is needed to achieve optimal effects, which in turn has a great number of side effects [68]. Since then, researchers have developed a CD11B agonist (ADH-503) that can achieve higher rates of myeloid cell depletion as compared to previous CD11B blocking therapies. This drug also elicits tumor cell regression and T cell infiltration in murine models of pancreatic ductal adenocarcinoma [69].

There are also a number of options to target MDSCs that are being tested in clinical trials (Table 1). Some of these include:

1. JAK/STAT Pathway—The JAK/STAT pathway is essential in promoting the expansion of MDSCs and regulating their immunosuppressive functions. STAT3 can induce the expression of immunosuppressive factors such as Nox2 and Arginase. Axitinib is a selective inhibitor of vascular endothelial growth factor receptor (VEGFR) and studies have shown that it can decrease the number of mMDSCs in a murine melanoma model and also changes their phenotype from immunosuppressive cells to antigen-presenting cells that can activate cytotoxic T cells [70]. STAT3 inhibition by Sunitinib has also shown efficacy in reducing the numbers of MDSCs and promoting tumor regression in murine renal cell carcinoma [71].

2. CXCR2 Signaling Pathway—The CXCR2 pathway is essential for regulating neutrophil recruitment and it is an attractive target for therapies aimed at reducing MDSCs within the TME. CXCL8 is the most widely studied CXCR2 ligand and studies have shown that blocking this signaling axis can lead to TNBC tumor cell death [72] and a downregulation of IL8 by proteosome inhibition.

3. Arginase Inhibitors—Arg1 is a known immunosuppressive factor produced by MDSCs to reduce nutrients within the TIME that aid T cell survival and expansion. The arginase inhibitor, CB-115 has shown therapeutic efficacy in multiple murine tumors models, including the 4T1 model of breast cancer, by reducing the numbers of tumor-infiltrating myeloid cells and encouraging anti-tumor CD8+ T and NK cell responses [66].

4. MDSC differentiation—Drugs such as Decitibine, a DNMT1 inhibitor, can not only target tumor cells but can also promote the conversion of MDSCs into antigen-presenting cells. In vitro, these myeloid cells produce lower amounts of immunosuppressive and pro-angiogenic factors such as Il3, Il10, VEGF. Adoptive transfer of these cells into tumor-bearing mice can promote tumor rejection in mice [73].

5. Notch Pathway—The Notch signaling pathway is often upregulated in breast cancers. It plays a critical role in breast cancer progression [74] by promoting the survival of TICs and leading to chemoresistance. MDSC-induced Notch signaling has been shown to drive the survival and proliferation of tumor-initiating cells in murine models of breast cancer [54]. Gamma secretase inhibitors that target this pathway are currently being tested in multiple preclinical and clinical studies although these can induce goblet cell metaplasia and toxicity [75].

6. Estrogen signaling in MDSCs—Preclinical studies have shown that bone marrow myelopoiesis depends on STAT3 activation by estrogen receptor alpha (ERα). Depleting ERα by using anti-estrogen drugs has shown to decrease gMDCS and mMDSC recruitment on breast tumor-bearing mice and can elicit a T cell driven anti-tumor immune response [76,77].

There are ongoing and completed clinical trials, as shown in Table 1, that target some of the above-mentioned pathways. Recently, a CSF1R inhibitor, Pexidartinib, was approved by the FDA to treat tenosynovial giant cell tumors [74] but the efficacy for any of these drugs in TNBC remains to be seen.

## 11. Conclusions

There is no “one size fits all” approach to immunotherapy. While targeting immunosuppressive myeloid cells has led to reduction in tumor volumes and increased survival in preclinical studies in murine models, these drugs as single agents have failed to live up to their promises in human clinical trials. Due to the innate complexity of the TIME, depleting one cell type usually leads to a compensatory increased recruitment of other immunosuppressive myeloid cells that benefit tumor cell survival and proliferation [52].

An important issue that needs to be taken into consideration when depleting immunosuppressive cells in the TIME using targeted therapy is to identify those tumors which are highly dependent on the target for survival and proliferation. The levels of PD1-PDL1 expression via IHC are important tools in selecting the cohort of patients most likely to benefit from the checkpoint blockade therapy [78]. This highlights the urgent need for biomarkers to identify TAMs/MDSCs that can be used in clinical assays to provide personalized treatments to patients without subjecting them to unnecessary treatments that may not provide any therapeutic benefits and lead to a slew of adverse events. Another important issue as we further develop and refine myeloid cell targeting drugs is to identify targets that are specific to those particular cells and do not cause injury to healthy tissues/organs. For example, CSF1R is ubiquitously expressed by a number of organ-specific macrophages including the Kupffer cells in the liver and clinical trials using anti-CSF1R drugs have been shown to cause an elevation in liver enzymes in patients that is usually reversable upon treatment cessation. Similarly, while low-dose chemotherapy such as cisplatin and gemcitabine have shown to reduce the number of MDSCs within the TIME other studies have shown that certain chemotherapy drugs such as cyclophosphamide can lead to an increase in MDSC subpopulations in TC-1 tumor-bearing mice [79].

A number of monotherapies targeting myeloid cells have failed to show significant therapeutic benefits. While reducing the numbers of immunosuppressive myeloid cells is a potential strategy, there need to be other ways in which the adaptive immune system can be targeted to promote tumor cell killing. Clinical trials in a number of solid cancers including TNBC are ongoing that combine a CSF1R inhibitor with PD1-targeting drugs in the hopes that a synergistic effect might lead to an increased infiltration of ant-tumor T cells, B Cells and NK cells that can promote long-term regression of these tumors and even prevent distant recurrences.

In conclusion, TAMs and MDSCs are key immunosuppressive players within the TIME that promote tumor cell survival, metastasis and inhibit the adaptive arm of the immune system including T cells and NK cells that can target and kill these tumor cells. Leveraging effective preclinical models has led to an improved understanding of these cell types. On one hand, technologies such as single-cell RNA sequencing have enabled a better understanding of the heterogeneity in TAMs and MDSCs and potentially may lead to the identification of specific druggable targets on these cells. On the other hand, we now also understand the need to refine current therapeutic regimens to achieve the full treatment benefits. The future of cancer therapy now lies in the hands of the old guard and the new, i.e., combining standard-of-care regimens with new immunotherapy approaches. Bringing these two approaches together can lead to the breakthroughs we urgently need to overcome the hurdles of this disease.

## Figures and Tables

**Table 1:** Drugs targeting TAMs and myeloid-derived suppressor cells (MDSCs) in clinical trials for breast cancer.

Target	Drug	In Combination with	Phase	Clinical Trial Identifier
CSF1R/CSF1	Ipatasertib	Atezo or pac or nab-Paclitaxel	1b	NCT03800836 *
	Pexidartinib	nAB-Paclitaxel Eribulin	1b 1a/1b	NCT01525602 NCT01596751
	LY3022855	Durvalumab OR tremelimumab	1b	NCT02718911
	Emactuzumab	Selicrelumab	1b	NCT02760797
	Lacnotuzumab	Spartalizumab	1b/2	NCT02807844
CCL2/CCR2	Carlumab		1b	NCT01204996
CD47/SIRPα	IBI322	Pembrolizumab	1a/1b	NCT04328831 *
	ALX148	Pembrolizumab, trastuzumab or chemo	1	NCT03013218 *
	Hu5F9-G4		1	NCT02216409
ARGINASE	INCB001158		1	NCT02903914 *
MDSC DIFFERENTIATION	Decitabine		1	NCT00030615
MDSC SURVIVAL	Entinostat	Ipilimumab and Nivolumab	1	NCT02453620 *

Asterisk * indicates clinical trials that are currently ongoing.
